# Human Bocavirus in Brazil: Molecular Epidemiology, Viral Load and Co-Infections

**DOI:** 10.3390/pathogens9080645

**Published:** 2020-08-10

**Authors:** Fábio Correia Malta, Rafael Brandão Varella, Maria Angelica Arpon Marandino Guimarães, Marize Pereira Miagostovich, Tulio Machado Fumian

**Affiliations:** 1 Laboratory of Comparative and Environmental Virology, Oswaldo Cruz Institute, Oswaldo Cruz Foundation, Rio de Janeiro 21040-360, Brazil; fabio.malta@ioc.fiocruz.br (F.C.M.); marizepm@ioc.fiocruz.br (M.P.M.); 2 Department of Infectious and Parasitic Diseases, School of Medicine, Clementino Fraga Filho University Hospital, Federal University of Rio de Janeiro, Rio de Janeiro 21941-590, Brazil; maamguimaraes@globo.com; 3 Department of Microbiology and Parasitology, Institute Biomedical, Federal Fluminense University, Niterói 24210-130, Brazil; rvarella@id.uff.br

**Keywords:** acute gastroenteritis, human bocavirus, co-infections, viral load, genotyping, Brazil

## Abstract

Human bocavirus (HBoV) is an emerging virus and has been detected worldwide, especially in pediatric patients with respiratory and gastrointestinal infection. In this study, we describe HBoV prevalence, genotypes circulation and DNA shedding, in stool samples from children up to two years of age in Brazil. During 2016 and 2017, 886 acute gastroenteritis (AGE) stool samples from ten Brazilian states were analyzed by TaqMan^®^-based qPCR, to detect and quantify HBoV. Positive samples were genotyped by sequencing the VP1/2 overlap region, followed by phylogenetic analysis and co-infections were accessed by screening other gastroenteric viruses. HBoV was detected in 12.4% (n = 110) of samples, with viral load ranging from 1.6 × 10^2^ to 1.2 × 10^9^ genome copies per gram of stool. From these, co-infections were found in 79.1%, and a statistically lower HBoV viral load was found compared to viral loads of rotavirus, norovirus and adenovirus in double infected patients (*p* < 0.05). No significant differences were found between HBoV viral load in single or co-infections, age groups or genotypes. Phylogenetic analysis identified the circulation of HBoV-1 in 38%, HBoV-2 in 40% and HBoV-3 in 22%. Continuous HBoV monitoring is needed to clarify its role in diarrhea disease, especially in the absence of classic gastroenteric viruses.

## 1. Introduction

Acute gastroenteritis (AGE), a preventable disease, still figures as a leading cause of death in young children and contributes significantly to childhood morbidity, especially in low-income countries [1,2,3]. Among the main viral agents associated with pediatric AGE, rotavirus A (RVA), norovirus and human adenovirus (HAdV) figure as the main etiological agents worldwide [3,4,5]. However, the etiology of a significant number of AGE cases (up to 40%) remains undermined, especially in developing countries [6,7]. A diagnostic gap as large as a third of all AGE specimens has been estimated [8], suggesting that less common viral agents could play a role in causing the disease. 

Human bocaviruses (HBoV), members of the *Parvoviridae* family and genus *Bocaparvovirus* were first described in 2005 in respiratory swabs from children with lower respiratory tract infections, and designated as HBoV-1 [9]. After its first discovery, three other HBoV related viruses (HBoV-2, 3 and 4) were identified in human stool samples from children with AGE illness [10,11,12]. However, as these genotypes are often co-detected with other gastroenteric viruses, it is still unclear whether the linking of these genotypes as causative agents of gastroenteritis [13,14,15,16,17]. 

HBoV are small (20 nm) non-enveloped viruses with a single-stranded DNA genome of approximately 5 kb nucleotides (nt) in length; its genome is organized into three open reading frames (ORFs). ORF1 encodes for the non-structural protein (NS1), ORF2 encodes for the two capsid proteins (VP1–2), and ORF3 encodes for the nucleoprotein (NP1) [9,18,19]. Currently, based on the genomic analysis of the two major structural proteins (VP1–2), HBoV is divided into four species or genotypes (HBoV-1 to 4) [12]. HBoV genotypes are globally distributed, and have been single or co-detected in AGE cases, with incidence varying from 0.5 to 25%, especially in children younger than two years old [10,16,19,20,21]. 

In Brazil, there is still a lack of data concerning the role of HBoV as causative agents of AGE, especially concerning the detection of HBoV and simultaneous investigations of multiple other enteric viruses, as well as HBoV shedding in stool specimens. Therefore, in the present study, we aimed to investigate HBoV among AGE cases from children in Brazil over a period of two years (2016–2017). In order to access the frequency of single or co-infection, HBoV and other major gastroenteric viruses, such as RVA, norovirus GI and GII, HAdV, sapovirus and astrovirus were also tested by RT-qPCR or qPCR assays. We also compared HBoV DNA shedding among single and co-infected samples, and determined HBoV molecular epidemiology during the period, by sequencing the partial VP1 gene of positive samples. Additionally, we investigated the correlation between HBoV fecal viral load and other features, such as age groups and HBoV genotypes.

## 2. Materials and Methods

### 2.1. Stool Collection and Ethics Aspects

This study included stool samples that were collected between January 2016 and December 2017, from children up to two years of age, with symptoms of AGE. For instance, AGE was characterized as ≥three liquid/semi liquid evacuations in a 24-h period. AGE stool samples were collected by sentinels’ sites at States Central Laboratories, from ten Brazilian states and three regions (Southern, Southeastern, and Northeastern) and sent to the Laboratory of Comparative and Environmental Virology that houses the Regional Rotavirus Reference Laboratory (RRRL) at Oswaldo Cruz Institute.

The RRRL takes part in the ongoing national viral AGE surveillance program coordinated by General Coordination of Public Health Laboratories, Brazilian Ministry of Health covering diagnosis, surveillance and molecular epidemiology of circulating viruses. The surveillance is performed through a hierarchical network, in which samples are provided by medical request in hospitals and health centers, monitored by the Brazilian Unified Health System (SUS). Patients’ data were maintained anonymously and securely. This study is approved by the Ethics Committee of the Oswaldo Cruz Foundation (FIOCRUZ), number CAAE: 94144918.3.0000.5248.

### 2.2. Nucleic Acid Extraction 

Viral nucleic acids (DNA and RNA viruses) were purified from 140 μL of clarified stool suspension (10% w/v) prepared with Tris-calcium buffer (pH = 7.2). Samples were subjected to an automatic nucleic acid extraction procedure using a QIAamp^®^ Viral RNA Mini kit (QIAGEN, Valencia, CA, USA), and a QIAcube^®^ automated system (QIAGEN, Valencia, CA, USA), according to the manufacturer’s instructions, and eluted in 60 µL of AVE elution buffer. The isolated nucleic acid was immediately stored at −80 °C until the molecular analysis. In each extraction procedure, RNAse/DNAse-free water was used as negative control.

### 2.3. HBoV Detection and Quantification 

HBoV were detected and quantified by using a TaqMan^®^-based qPCR protocol, as previously described [22]. Five primers covering the untranslated region and the beginning of the NS1 of the HBoV genome was used with a 5′-FAM and 3′-MGB-labeled probe, designed from a fully conserved region among all HBoV genotypes. Moreover, qPCR reactions were performed with 5 µL of the extracted DNA, in a volume of 25 µL, using the Applied Biosystems^®^ 7500 Real-Time PCR System and TaqMan Universal Master Mix (Applied Biosystems, Foster City, CA, USA), with final concentrations of 0.6 µM and 0.3 µM of primers and probe, respectively. 

To estimate HBoV viral load, a standard curve prepared by six 10-fold serial dilutions (10^6^–10^1^ genome copies (GC) per reaction) of a double-stranded DNA fragment (gBlock^®^ Gene Fragment, Integrated DNA Technologies, Coralville, IA, USA) containing the HBoV amplification region sequence was used in each qPCR reaction. All samples that crossed the threshold line showing a characteristic sigmoid curve with a cycle threshold (Ct) value <40 were regarded as positive. All runs included negative and non-template controls (NTC), to ensure the correct interpretation of the results throughout the study. HBoV viral load was expressed as GC per gram (GC/g) of stool that also corresponds to GC per ml of starting stool material.

### 2.4. Molecular Characterization and Genotyping

Nested PCR reactions targeting the VP1/2 region were performed for HBoV sequencing. Primers AK-VP-F1/R1 and AK-VP-F2/R2 were used in the first and second round, respectively, generating a 576 base pairs (bp) amplicon, as previously described by Kapoor et al. [12] The amplicons obtained were purified using a QIAquick PCR Purification Kit (QIAGEN, Valencia, CA, USA), following the manufacturer’s recommendations. Sequencing reactions were performed using both forward and reverse primers with the BigDye™ Terminator v. 3.1 Cycle Sequencing Kit (Applied Biosystems, Foster City, CA, USA). Subsequently, reactions were purified and sent to the FIOCRUZ Institutional Sequencing Platform (PDTIS), where they were run on an ABI Prism 3730*xl* genetic analyzer (Applied Biosystems).

### 2.5. Phylogenetic Analysis

HBoV consensual sequences were obtained after nucleotide (nt) alignment and edition using Geneious prime (Biomatters Ltd., Auckland, New Zealand), and genotypes were confirmed in terms of closest homology sequence, using Basic Local Alignment Search Tool (BLAST). Phylogenetic trees were constructed using the maximum likelihood method (2000 bootstrap replications for branch support) in MEGA X v. 10.1.7 [23], with HBoV reference sequences obtained from the National Center for Biotechnology Information (NCBI) database. Nucleotide sequences obtained in this study were submitted to NCBI GenBank (accession numbers: MN648223 to MN648316 and MN652577 to MN652584).

### 2.6. Gastroenteric Viruses Detection and Quantification

In order to investigate viral co-infections, all HBoV-positive samples were tested for other major gastroenteric viruses—RVA, HAdV, norovirus GI and GII, sapovirus and astrovirus. For viral detection and quantification, TaqMan^®^-based qPCR reactions were used with primers and probes and the same reactions conditions as previously described [24,25,26,27,28]. Viral load was determined by using standard curves generated from ten-fold serially diluted gBlocks dsDNA fragments containing the target qPCR region for each virus. All the viral load data are present here as GC/g of stool sample.

### 2.7. Statistical Analysis 

Statistical analyses were performed using GraphPad Prism v. 8.4.1 (GraphPad Software, San Diego, CA, USA). Box-and-whisker plots were produced to illustrate differences between medians with interquartile ranges. Mann–Whitney U test was used for comparison of viral load values between HBoV single and co-infection, age groups, genotypes, and among dual co-infected samples. Chi-square and Fisher’s exact tests were used for analyzing categorical characteristics in contingency tables. For all analyses, a *p*-value < 0.05 was considered to be statistically significant.

## 3. Results

### 3.1. HBoV Epidemiology

**Figure 1:**
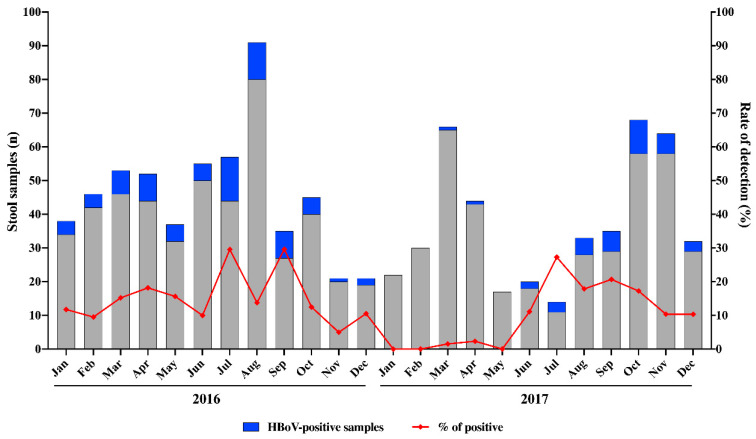
Monthly distribution of tested acute gastroenteritis stool samples, human bocavirus (HBoV)-positive samples and detection rates in Brazil, 2016–2017.

During the two-year period of this study (2016–2017), we analysed a total of 886 stool samples from symptomatic children aged ≤2 years. Overall, HBoV was detected in 110 samples (12.4%) by qPCR. Of these, HBoV was detected in 15.3% (73/478) and in 9.1% (37/408) of samples collected in 2016 and 2017, respectively. We detected HBoV year-round without a marked seasonality. However, higher HBoV circulation was observed between July and September (Figure 1). Except for three months that HBoV was not detected, monthly detection rates varied from 5% to 29.6% in 2016, and from 1.5% to 27.3% in 2017 (Figure 1). 

In our study, HBoV was detected in 14.1% and 10.5% of samples from male and female, respectively, without statistical significance (*p* > 0.05). Table 1 shows the epidemiological features of HBoV-positive cases, co-infections and a detailed analysis of HBoV infections by age group, gender, genotypes and regions. In regard to age groups, we detected HBoV in 9%, 11.2% and 15.7% of samples, among children aged from 0 to ≤6, >6 to ≤12 and >12 to ≤24 months old, respectively. We found a statistically higher HBoV positivity in stool samples collected from children aged >12 to ≤24 months old, compared to the other age groups (*p* = 0.043).

Concerning regional analysis, HBoV annual detection rates varying from 5.1% to 17.5% in South and Northeast regions, respectively (Table 1). We also compared HBoV detection rates between eight states from Northeastern and Southeastern Brazil, characterized as tropical climate, and two states from Southern (Santa Catarina e Rio Grande do Sul), characterized as subtropical climate pattern. A higher HBoV positivity was identified in states located in the tropical area (13.8%) compared to states located in the subtropical area (10.4%), however, no significant difference was observed comparing the detection rates between the two regions (*p* > 0.05) (Table 1).

### 3.2. Viral Load and Co-Infections

In order to access HBoV single or co-infections, we investigated the presence of the major gastroenteric viruses among the HBoV-positive samples (n = 110). We found that in 20.9% of samples (n = 23), HBoV was detected as single infection, whilst 79.1% of samples (n = 87) were co-infected with one or more viral agent (RVA, HAdV, norovirus GI and GII, sapovirus and astrovirus) (Table 1). Among the co-infections, the majority (77%) was characterized as dual infections. Three or four viral pathogens were detected in 18.4% (n = 16) and 4.6% (n = 4) of samples, respectively.

**Figure 2:**
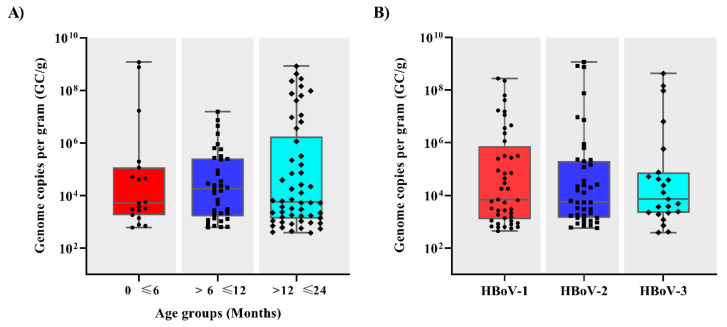
Human bocavirus (HBoV) viral load expressed as genome copies per gram of stool (GC/g) among different age groups (**A**) and genotypes (**B**) in Brazil. Box-and-whisker plots show the first and third quartiles (equivalent to the 5th and 95th percentiles), the median (the horizontal line in the box), and range of HBoV viral load concentrations.

Regarding DNA quantification, qPCR results demonstrated that HBoV viral load varied broadly among patients, ranging from 3.9 × 10^2^ to 1.2 × 10^9^ GC/g of stool samples, with a median of 6.7 × 10^3^ GC/g. No significant differences were observed between HBoV viral load and age groups (Figure 2A) or among HBoV-1, 2 or 3 genotypes (*p* > 0.05) (Figure 2B).

**Figure 3:**
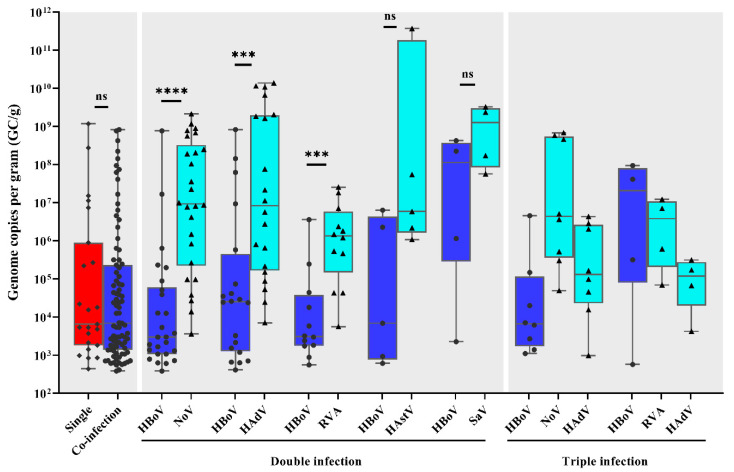
Human bocavirus (HBoV) viral load among single and co-infections, and analysis of enteric viruses’ viral load among HBoV-positive co-infected samples. Box-and-whisker plots show the first and third quartiles (equivalent to the 5th and 95th percentiles), the median (the horizontal line in the box), and range of HBoV viral load concentrations, expressed as genome copies per gram of stool (GC/g). ns, not significant; *** *p* ≤ 0.001; **** *p* ≤ 0.0001.

Comparing HBoV viral load found in single and co-infections, values ranged from 4.4 × 10^2^ to 1.2 × 10^9^ GC/g and 3.9 × 10^2^ to 8.3 × 10^8^ GC/g, with median values of 6.5 × 10^3^ GC/g and 6.9 × 10^3^ GC/g, respectively (*p* > 0.05) (Figure 3). Among the co-infections, we also compared the viral load of HBoV with viral load found of other identified viruses. HBoV viral load values were significantly lower compared to norovirus, HAdV and RVA viral loads in dual infections of HBoV with one of those viruses (*p* < 0.05) (Figure 3).

### 3.3. Molecular Characterization

**Figure 4:**
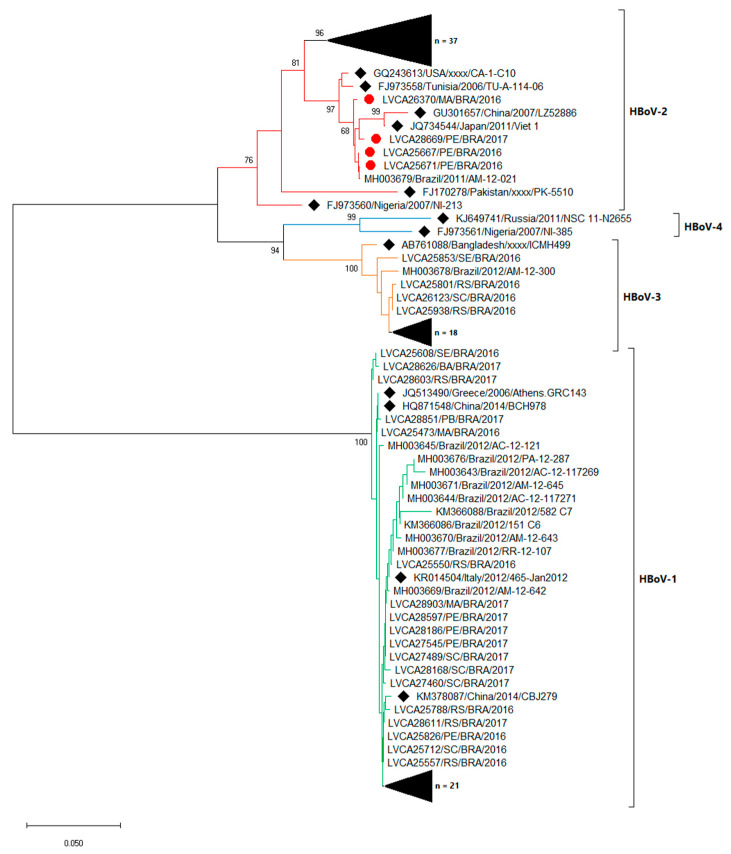
Phylogenetic analyses based on VP1 nucleotide (nt) sequences of circulating Brazilian human bocavirus (HBoV) strains. Reference strains, marked with a black filled diamond, were downloaded from GenBank and labelled with their accession number followed by country, year and register number. Strains obtained are shown as per LVCA followed by the internal register number, state, country and year of collection (i.e., LVCA28597/PE/BRA/2017), and the red filled circle represents sequences with a three-nt insertion. Neighbour-joining phylogenetic tree was constructed with MEGA X software and bootstrap tests (2000 replicates), based on the Kimura two-parameter model. Bootstrap values above 70% are given at branch nodes.

We successfully sequenced 92.7% of positive samples (102/110). By molecular characterization, we identified the circulation of HBoV-1, 2 and 3 during the study period. The phylogenetic tree of partial region of VP1 demonstrated that HBoV-1, 2 and 3 were characterized in 38% (n = 39), 40% (n = 41) and 22% (n = 22) of samples, respectively. From sequences obtained in 2016 and 2017, HBoV-1 was identified in 31.5% and 59.5%, HBoV-2 in 39.7% and 35.1% and HBoV-3 in 28.8% and 5.4%, respectively (Figure 4).

Among HBoV-1, 2 and 3 strains detected, nt similarity varied from 98.9% to 100%, 94.1% to 100% and 97.1% to 100%, respectively. HBoV-1 sequences were genetically related (>99% of nt identity) to previous strains detected in Brazil (KY882298, KX826925 and MF034122), as well as for HBoV-2 isolated sequences (MF034103 and KX826932) and HBoV-3 sequences (MF156864, MG953833 and KX826937). Four HBoV-2 sequences showed an amino acid insertion (Gln) at position 18 of VP2, in relation to the HBoV-2 prototype (FJ170278, nt position 3397–3399). These four samples were isolated from Northeastern Brazil, three from the same state, Pernambuco, and one from Maranhão state. Another four HBoV-2 sequences showed one amino acid deletion (Gly) at position 23 of VP2, in relation to the same HBoV-2 prototype (nt position 3405–3407). These four sequences were isolated from samples collected between August and September 2016 from Rio Grande do Sul state, Southern Brazil. 

## 4. Discussion

In the present study, we determined HBoV prevalence among children up to two years old in Brazil, as well as co-infections, fecal viral load and the characterization of circulating genotypes. Overall, HBoV was detected in 12.4% of 886 AGE stool samples between 2016 and 2017, and co-infections with one or multiple gastroenteric viruses were detected in the majority of the HBoV-positive samples.

Our results corroborate with data from few studies conducted in Brazil that have investigated HBoV in stool samples from AGE cases. Recently, two studies with hospitalized children presenting AGE symptoms from Northern Brazil reported HBoV detection rates of 24% [21] and 12.2% [29]. Albuquerque et al. [30] found HBoV in 2% of stool samples (n = 705) from inpatient and outpatient children with AGE symptoms. In that study, children were older (median age of 3.5 years) compared to our study (children <2 years old), explaining the lower positivity rate, and in fact, the authors demonstrated that 78.6% of HBoV-positive samples were among children <2 years old. A study conducted in Central-Western Brazil during 1994 and 2004 analyzed 762 AGE samples from children under five years and found HBoV in a positivity rate of 5.8% [31]. The highest detection rate of HBoV in Brazil (42%) was described by Campos et al. [32] in Northeastern Brazil, among 105 stool samples from children with AGE. 

Other studies performed in Brazil have accessed HBoV DNA in stool samples from patients with underlying medical conditions, such as HIV infected and immunosuppressed transplanted patients, showing detection rates varying from 13% to 21.4% [33,34,35]. Worldwide, several studies have detected HBoV in hospitalized children and outpatients with AGE at rates between 3.5% and 19.3%, in countries such as China, Spain, Chile, Pakistan and South Africa [14,36,37,38,39,40]. A recent study reviewing reports on HBoV detection in individuals with AGE in Africa between 2005 and 2016 found that HBoV prevalence was 13% [41], similar to HBoV-positivity found in our study. 

Regarding age groups, we found significant higher detection of HBoV in children aged >6 to 24 months old, compared to younger children up to 6 months. It has been described that HBoV most affected age group are children up to 24-months-old [17,19,37,42]. A large study performed in South Africa between 2009 and 2015 with 3765 stool specimens collected from hospitalized children for AGE demonstrated that the majority (92%) of HBoV-positive cases were children <2 years old [20]. Furthermore, the lower rates of HBoV infection among children aged <6 months found in our study and reported elsewhere are likely due to early protection by the maternal antibodies [43,44,45], and also because usually after six-months-old, children are more exposed to viral infections within childcare centers. 

In our study, HBoV was detected year-round without marked seasonality. However, we observed higher detection rates between July and September, corresponding to winter season (June 21st to September 22nd) in Brazil. Despite not being well-established, some studies have demonstrated higher detection rates of HBoV in winter [46,47,48,49]. Our results also support findings widely reported related to the high percentage of co-infections among HBoV-positive samples, and here, we found co-infections in nearly 80% of these samples. This high percentage could be associated to multiple gastroenteric viruses tested in our study—RVA, HAdV, norovirus GI and GII, sapovirus and astrovirus. Previously in Brazil, Soares et al. [21] identified co-infections between HBoV and RVA in 50% of cases. In China, two studies described co-infections of HBoV with either RVA or norovirus in 64% of cases [37], and in 77.6% of cases co-infected with RVA, norovirus, astrovirus, or enteric HAdV [38]. Other studies demonstrated high percentages of co-infections, varying from 98% to 100% of the analyzed samples [14,16]. More recently, Netshikweta et al. [20], screening for enteric viruses, bacteria and parasites, detected HBoV as a sole agent in just 0.9% among 1654 studied cases. Interestingly, the same pattern of high co-infection rates is observed in HBoV-positive samples from acute respiratory infections (ARI). During a 3-year study of HBoV in 1015 outpatients and inpatients with symptoms of ARI in Brazil, co-infections with at least one additional respiratory virus were detected in 72.9% (35/48) of HBoV-positive patients [50].

Evaluating HBoV DNA shedding, we detected a broad range of viral loads, varying from 3.9 × 10^2^ a 1.2 × 10^9^ GC/g of stool samples. Unexpectedly, the median viral load of HBoV in stool of children with or without coinfection were almost identical. Few studies have reported HBoV shedding in stool samples from AGE cases. Similar HBoV viral load ranges in stool from patients with AGE were described in China, varying from 1.7 × 10^2^ to 4.3 × 10^9^ GC/mL of stool [51] and Finland, from <10^3^ to 10^9^ GC/mL [22]. In a case-control study from China, HBoV shedding varied from 1.5 × 10^1^ to 9.9 × 10^8^ and from 2.9 × 10^1^ to 4.5 × 10^2^ GC/mL of stool, among cases and controls, respectively, but no statistical difference was observed between the groups [52]. Similar findings were also reported by Cheng et al. [37] and Nawaz et al. [53] in case-controls studies, where no significant differences were identified in viral loads between the two groups.

To the best of our knowledge, this is the first study comparing the viral load of HBoV and other gastroenteric viruses in co-infected patients. Interestingly, we noted that for dual co-infections of HBoV with one of the classical diarrheic viruses (norovirus, HAdV or RVA), a statistically lower viral load of HBoV was found. The clinical significance of these differences is unclear, but as high viral load detection suggests active replication, it is likely that HBoV has a secondary or synergistic role in these co-infection cases. For ARI, quantitative HBoV DNA analysis is one of the recommended diagnostic approaches of primary infection, along with serology and mRNA detection [54]. Regarding HBoV in patients with ARI, Neske et al. [55] demonstrated that children shedding HBoV in stool samples had significantly higher HBoV load in nasopharyngeal aspirates (NPA), compared to children that tested HBoV-negative in stool samples. Another study found that rates of diarrhea were significantly higher in patients with very high HBoV viral loads in their NPAs [50]. It is worth mentioning that during the ongoing pandemic of respiratory disease caused by severe acute respiratory syndrome coronavirus 2 (SARS-CoV-2), many studies have reported an incidence rate of diarrhea or viral shedding in stool, ranging from 2% to 59% of cases with high viral loads [56,57,58], along with the replication of SARS-CoV-2 in human gut enterocytes [59]. Similar to HBoV infection that causes ARI and AGE, and although the pathogenesis and transmission of both viruses are not entirely understood, the findings suggest active viral replication within both respiratory and gastrointestinal tracts.

Our study presents several limitations. We did not evaluate the presence of mRNA in stool samples, and we did not search for enteropathogens other than gastroenteric viruses, for instance, bacteria and parasites, that likely play a role in AGE clinical cases. An additional limitation is that, as our study only included symptomatic patients, it was not possible to evaluate HBoV viral load differences among cases and health controls. Further studies on these topics are warranted.

Concerning HBoV genotypes circulation in Brazil, we identified genotypes 1, 2 and 3 in AGE cases, without significant difference of viral load among them. Many studies have reported the association of HBoV-2, 3 and 4 with AGE symptoms, whilst HBoV-1 has been more associated with respiratory infections [13,54,60,61]. In Southeastern Brazil, Santos et al. [62] firstly demonstrated the circulation of HBoV-2 (20.8%) and HBoV-3 (0.6%) in stool samples from patients with AGE. More recently, in Northern Brazil, two studies have reported the circulation of HBoV-1, 2 and 3 in stool samples from children hospitalized for AGE, and both found a predominance of genotype 1 (94.8% and 75%) [21,29]. In line with our findings, studies performed elsewhere have predominantly detected genotypes 1–3 in AGE cases [13,63,64,65], and HBoV-4 has been rarely detected, or detected in lower prevalence compared to other genotypes [12,39,52,60,63,66]. In Thailand, Khamrin et al. [67] reported a higher detection of HBoV-1 (64.7%) in AGE samples, followed by HBoV-2 and 3, and just one sample was characterized as HBoV-4. Nevertheless, other studies have found HBoV-2 as the most frequently detected genotype in AGE cases, as described in China, Australia and South Korea [10,52,63]. HBoV-2 infection was demonstrated to be a risk factor of AGE in children <5 years old [66], despite the fact that some studies have considered HBoV to be a bystander agent of diarrheic disease [15].

In conclusion, over a period of two years (2016–2017), we detected HBoV in 12.4% of AGE stool samples from children up to two years old from ten Brazilian states, which represents almost half of the country’s population. No differences between HBoV DNA shedding and genotypes, age groups or single and co-infections were observed. We detected high rates of viral co-infections among HBoV-positive samples, and found a significant inverse correlation between norovirus, RVA or HAdV viral loads and HBoV DNA shedding in dual co-infections. Nevertheless, some HBoV single infected-patients were detected as shedding high viral loads (up to 10^9^ GC/g of stool) suggesting active replication. Future studies are warrant to determine the role of HBoV in causing acute diarrhea, especially when the classical gastroenteric viruses are not identified.

## Figures and Tables

**Table 1:** Frequency of human bocavirus (HBoV) detection from diarrheic stool samples through laboratory-based surveillance according to single and co-infection, age group, gender, genotypes and by region in Brazil.

HBoV Detection			Positive/Tested (%)
	Single-Infection		23/886 (20.9)
	Co-Infection		87/886 (79.1)
**Co-infections**	HBoV + norovirus		26/110 (23.7)
	HBoV + HAdV		20/110 (18.2)
	HBoV + RVA		12/110 (11)
	HBoV + astrovirus		5/110 (4.5)
	HBoV + sapovirus		4/110 (3.6)
	HBoV + norovirus + HAdV		8/110 (7.3)
	HBoV + RVA + HAdV		4/110 (3.6)
	HBoV + RVA + norovirus		3/110 (2.7)
	HBoV + norovirus+ sapovirus		1/110 (0.9)
	HBoV + RVA + norovirus+ HAdV		3/110 (2.7)
	HBoV + norovirus+ HAdV + astrovirus		1/110 (0.9)
**Age groups (months)**			
0 to ≤6			19/212 (9)
>6 to ≤12			37/331 (11.2)
>12 to ≤24			54/343 (15.7) *
**Gender**			
Male			69/488 (14.1)
Female			41/391 (10.5)
**Genotypes**			
HBoV-1			39/102 (38)
HBoV-2			41/102 (40)
HBoV-3			22/102 (22)
**Regions**	**2016**	**2017**	
Northeast	27/154 (17.5)	23/180 (12.7)	50/334 (14.9)
Southeast	9/70 (12.8)	4/31 (12.9)	13/101 (12.8)
South	37/254 (14.5)	10/197 (5.1) *	47/451 (10.4)

* *p* < 0.05, Chi-square and Fisher’s exact tests.

## References

[1] Lozano R., Naghavi M., Foreman K., Lim S., Shibuya K., Aboyans V., Abraham J., Adair T., Aggarwal R., Ahn S.Y. (2012). Global and regional mortality from 235 causes of death for 20 age groups in 1990 and 2010: A systematic analysis for the Global Burden of Disease Study 2010. Lancet.

[2] Walker C.L.F., Rudan I., Liu L., Nair H., Theodoratou E., Bhutta Z.A., O’Brien K.L., Campbell H., Black R.E. (2013). Global burden of childhood pneumonia and diarrhoea. Lancet.

[3] Troeger C., Forouzanfar M., Rao P.C., Khalil I., Brown A., Reiner R.C., Fullman N., Thompson R.L., Abajobir A., Ahmed M. (2017). Estimates of global, regional, and national morbidity, mortality, and aetiologies of diarrhoeal diseases: A systematic analysis for the Global Burden of Disease Study 2015. Lancet Infect. Dis..

[4] Wilhelmi I., Roman E., Sanchez-Fauquier A. (2003). Viruses causing gastroenteritis. Clin. Microbiol. Infect..

[5] Bányai K., Estes M.K., Martella V., Parashar U.D. (2018). Viral gastroenteritis. Lancet.

[6] Kotloff K.L., Nataro J.P., Blackwelder W.C., Nasrin D., Farag T.H., Panchalingam S., Wu Y., Sow S.O., Sur D., Breiman R.F. (2013). Burden and aetiology of diarrhoeal disease in infants and young children in developing countries (the Global Enteric Multicenter Study, GEMS): A prospective, case-control study. Lancet.

[7] Nicholson M.R., Van Horn G.T., Tang Y.-W., Vinjé J., Payne D.C., Edwards K.M., Chappell J.D. (2016). Using Multiplex Molecular Testing to Determine the Etiology of Acute Gastroenteritis in Children. J. Pediatr..

[8] Glass R.I., Bresee J.S. (2011). Astroviruses, Enteric Adenoviruses, and Other Gastroenteritis Viral Infections. Trop. Infect. Dis. Princ. Pathog. Pract..

[9] Allander T., Tammi M.T., Eriksson M., Bjerkner A., Tiveljung-Lindell A., Andersson B. (2005). Cloning of a human parvovirus by molecular screening of respiratory tract samples. Proc. Natl. Acad. Sci. USA.

[10] Arthur J.L., Higgins G.D., Davidson G.P., Givney R.C., Ratcliff R.M. (2009). A Novel Bocavirus Associated with Acute Gastroenteritis in Australian Children. PLoS Patho..

[11] Kapoor A., Slikas E., Simmonds P., Chieochansin T., Naeem A., Shaukat S., Alam M.M., Sharif S., Angez M., Zaidi S. (2009). A new bocavirus species in human stool. J. Infect. Dis..

[12] Kapoor A., Simmonds P., Slikas B., Li L., Bodhidatta L., Sethabutr O., Triki H., Bahri O., Oderinde B., Baba M. (2010). Human bocaviruses are highly diverse, dispersed, recombination prone, and prevalent enteric infections. J. Infect. Dis..

[13] Paloniemi M., Lappalainen S., Salminen M., Kätkä M., Kantola K., Hedman L., Hedman K., Söderlund-Venermo M., Vesikari T. (2014). Human bocaviruses are commonly found in stools of hospitalized children without causal association to acute gastroenteritis. Eur. J. Pediatr..

[14] Alam M.M., Khurshid A., Shaukat S., Sharif S., Suleman R.M., Angez M., Nisar N., Aamir U.B., Naeem M., Zaidi S.S.Z. (2015). Human bocavirus in Pakistani children with gastroenteritis. J. Med. Virol..

[15] Ong D.S.Y., Schuurman R., Heikens E. (2016). Human bocavirus in stool: A true pathogen or an innocent bystander?. J. Med. Virol..

[16] Rosa G.L., Libera S.D., Iaconelli M., Donia D., Cenko F., Xhelilaj G., Cozza P., Divizia M. (2016). Human bocavirus in children with acute gastroenteritis in Albania. J. Med. Virol..

[17] Zhou T., Chen Y., Chen J., Hu P., Zheng T., Xu X., Pei X. (2017). Prevalence and clinical profile of human bocavirus in children with acute gastroenteritis in Chengdu, West China, 2012–2013. J. Med. Virol..

[18] Schildgen O. (2013). Human Bocavirus: Lessons Learned to Date. Pathogens.

[19] Guido M., Tumolo M.R., Verri T., Romano A., Serio F., De Giorgi M., De Donno A., Bagordo F., Zizza A. (2016). Human bocavirus: Current knowledge and future challenges. World J. Gastroenterol..

[20] Netshikweta R., Chidamba L., Nadan S., Taylor M.B., Page N.A. (2019). Molecular epidemiology of human bocavirus infection in hospitalized children with acute gastroenteritis in South Africa, 2009–2015. J. Med. Virol..

[21] Soares L.S., Lima A.B.F., Pantoja K.C., Lobo P.S., Cruz J.F., Guerra S.F.S., Bezerra D.A.M., Bandeira R.S., Mascarenhas J.D.P. (2019). Molecular epidemiology of human bocavirus in children with acute gastroenteritis from North Region of Brazil. J. Med. Microbiol..

[22] Kantola K., Sadeghi M., Antikainen J., Kirveskari J., Delwart E., Hedman K., Söderlund-Venermo M. (2010). Real-Time Quantitative PCR Detection of Four Human Bocaviruses. J. Clin. Microbiol..

[23] Kumar S., Stecher G., Li M., Knyaz C., Tamura K. (2018). MEGA X: Molecular Evolutionary Genetics Analysis across Computing Platforms. Mol. Biol. Evol..

[24] Hernroth B.E., Conden-Hansson A.-C., Rehnstam-Holm A.-S., Girones R., Allard A.K. (2002). Environmental factors influencing human viral pathogens and their potential indicator organisms in the blue mussel, Mytilus edulis: The first Scandinavian report. Appl. Environ. Microbiol..

[25] Kageyama T., Kojima S., Shinohara M., Uchida K., Fukushi S., Hoshino F.B., Takeda N., Katayama K. (2003). Broadly reactive and highly sensitive assay for Norwalk-like viruses based on real-time quantitative reverse transcription-PCR. J. Clin. Microbiol..

[26] Oka T., Katayama K., Hansman G.S., Kageyama T., Ogawa S., Wu F.-T., White P.A., Takeda N. (2006). Detection of human sapovirus by real-time reverse transcription-polymerase chain reaction. J. Med. Virol..

[27] Zeng S.-Q., Halkosalo A., Salminen M., Szakal E.D., Puustinen L., Vesikari T. (2008). One-step quantitative RT-PCR for the detection of rotavirus in acute gastroenteritis. J. Virol. Methods.

[28] Dai Y., Xu Q., Wu X., Hu G., Tang Y., Li J., Chen Q., Nie J. (2010). Development of real-time and nested RT-PCR to detect astrovirus and one-year survey of astrovirus in Jiangmen City, China. Arch. Virol..

[29] Leitão G.A.A., Olivares A.I.O., Pimenta Y.C., Delgado I.F., Miagostovich M.P., Leite J.P.G., Moraes M.T.B. (2020). de Human Bocavirus genotypes 1 and 2 detected in younger Amazonian children with acute gastroenteritis or respiratory infections, respectively. Int. J. Infect. Dis..

[30] Albuquerque M.C.M., Rocha L.N., Benati F.J., Soares C.C., Maranhão A.G., Ramírez M.L., Erdman D., Santos N. (2007). Human Bocavirus Infection in Children with Gastroenteritis, Brazil. Emerg. Infect. Dis..

[31] De Sousa T.T., Souza M., Fiaccadori F.S., Borges A.M.T., da Costa P.S., das Dôres de Paula Cardoso D. (2012). Human bocavirus 1 and 3 infection in children with acute gastroenteritis in Brazil. Mem. Inst. Oswaldo Cruz.

[32] Campos G.S., Sampaio M.L.S., Menezes A.D.L., Tigre D.M., Costa L.F.M., Chinalia F.A., Sardi S.I. (2016). Human bocavirus in acute gastroenteritis in children in Brazil. J. Med. Virol..

[33] Portes S.A.R., Carvalho-Costa F.A., Rocha M.S., Fumian T.M., Maranhão A.G., de Assis R.M., da Penha Trindade Pinheiro Xavier M., Rocha M.S., Miagostovich M.P., Leite J.P.G. (2017). Enteric viruses in HIV-1 seropositive and HIV-1 seronegative children with diarrheal diseases in Brazil. PLoS ONE.

[34] Castro L.R.P., Calvet F.C., Sousa K.L., Silva V.P., Lobo P.S., Penha E.T., Guerra S.F.S., Bezerra D.A.M., Mascarenhas J.D.P., Pinheiro H.H.C. (2019). Prevalence of rotavirus and human bocavirus in immunosuppressed individuals after renal transplantation in the Northern Region of Brazil. J. Med. Virol..

[35] Costa B.C.L., Dábilla N.A.S., Almeida T.N., Fiaccadori F.S., Souza T.T., Cardoso D., Arantes A., Souza M. (2019). Human bocavirus detection and quantification in fecal and serum specimens from recipients of allogeneic hematopoietic stem cell transplantation: A longitudinal study. J. Med. Virol..

[36] Vicente D., Cilla G., Montes M., Pérez-Yarza E.G., Pérez-Trallero E. (2007). Human Bocavirus, a Respiratory and Enteric Virus. Emerg. Infect. Dis..

[37] Cheng W., Jin Y., Duan Z., Xu Z., Qi H., Zhang Q., Yu J., Zhu L., Jin M., Liu N. (2008). Human Bocavirus in Children Hospitalized for Acute Gastroenteritis: A Case-Control Study. Clin. Infect. Dis..

[38] Yu J.-M., Li D.-D., Xu Z.-Q., Cheng W.-X., Zhang Q., Li H.-Y., Cui S.-X., Miao-Jin, Yang S.-H., Fang Z.-Y. (2008). Human bocavirus infection in children hospitalized with acute gastroenteritis in China. J. Clin. Virol..

[39] Levican J., Navas E., Orizola J., Avendaño L.F., Gaggero A. (2013). Human Bocavirus in Children with Acute Gastroenteritis, Chile, 1985–2010. Emerg. Infect. Dis..

[40] Rikhotso M.C., Khumela R., Kabue J.P., Traoré-Hoffman A.N., Potgieter N. (2020). Predominance of Human Bocavirus Genotype 1 and 3 in Outpatient Children with Diarrhea from Rural Communities in South Africa, 2017–2018. Pathogens.

[41] Rikhotso M.C., Kabue J.P., Ledwaba S.E., Traoré A.N., Potgieter N. (2018). Prevalence of Human Bocavirus in Africa and Other Developing Countries between 2005 and 2016: A Potential Emerging Viral Pathogen for Diarrhea. J. Trop. Med..

[42] Lasure N., Gopalkrishna V. (2017). Molecular epidemiology and clinical severity of Human Bocavirus (HBoV) 1–4 in children with acute gastroenteritis from Pune, Western India. J. Med. Virol..

[43] Karalar L., Lindner J., Schimanski S., Kertai M., Segerer H., Modrow S. (2010). Prevalence and clinical aspects of human bocavirus infection in children. Clin. Microbiol. Infect..

[44] Hustedt J.W., Christie C., Hustedt M.M., Esposito D., Vazquez M. (2012). Seroepidemiology of Human Bocavirus Infection in Jamaica. PLoS ONE.

[45] Turin C.G., Ochoa T.J. (2014). The Role of Maternal Breast Milk in Preventing Infantile Diarrhea in the Developing World. Curr. Trop. Med. Rep..

[46] Smuts H., Hardie D. (2006). Human Bocavirus in Hospitalized Children, South Africa. Emerg. Infect. Dis..

[47] Lau S.K.P., Yip C.C.Y., Que T., Lee R.A., Au-Yeung R.K.H., Zhou B., So L., Lau Y., Chan K., Woo P.C.Y. (2007). Clinical and Molecular Epidemiology of Human Bocavirus in Respiratory and Fecal Samples from Children in Hong Kong. J. Infect. Dis..

[48] Lee J.I., Chung J., Han T.H., Song M., Hwang E. (2007). Detection of Human Bocavirus in Children Hospitalized because of Acute Gastroenteritis. J. Infect. Dis..

[49] Chhabra P., Payne D.C., Szilagyi P.G., Edwards K.M., Staat M.A., Shirley S.H., Wikswo M., Nix W.A., Lu X., Parashar U.D. (2013). Etiology of Viral Gastroenteritis in Children <5 Years of Age in the United States, 2008–2009. J. Infect. Dis..

[50] Proença-Modena J.L., Gagliardi T.B., Escremim de Paula F., Iwamoto M.A., Criado M.F., Camara A.A., Acrani G.O., Cintra O.A.L., Cervi M.C., de Paula Arruda L.K. (2011). Detection of Human Bocavirus mRNA in Respiratory Secretions Correlates with High Viral Load and Concurrent Diarrhea. PLoS ONE.

[51] Xu Z., Cheng W., Li B., Li J., Lan B., Duan Z. (2011). Development of a Real-Time PCR Assay for Detecting and Quantifying Human Bocavirus 2. J. Clin. Microbiol..

[52] Jin Y., Cheng W., Xu Z., Liu N., Yu J., Li H., Jin M., Li D., Zhang Q., Duan Z. (2011). High prevalence of human bocavirus 2 and its role in childhood acute gastroenteritis in China. J. Clin. Virol..

[53] Nawaz S., Allen D.J., Aladin F., Gallimore C., Iturriza-Gómara M. (2012). Human Bocaviruses Are Not Significantly Associated with Gastroenteritis: Results of Retesting Archive DNA from a Case Control Study in the UK. PLoS ONE.

[54] Christensen A., Kesti O., Elenius V., Eskola A.L., Døllner H., Altunbulakli C., Akdis C.A., Söderlund-Venermo M., Jartti T. (2019). Human bocaviruses and paediatric infections. Lancet Child. Adolesc. Health.

[55] Neske F., Blessing K., Tollmann F., Schubert J., Rethwilm A., Kreth H.W., Weissbrich B. (2007). Real-Time PCR for Diagnosis of Human Bocavirus Infections and Phylogenetic Analysis. J. Clin. Microbiol..

[56] Luo S., Zhang X., Xu H. (2020). Don’t Overlook Digestive Symptoms in Patients with 2019 Novel Coronavirus Disease (COVID-19). Clin. Gastroenterol. Hepatol..

[57] Wang W., Xu Y., Gao R., Lu R., Han K., Wu G., Tan W. (2020). Detection of SARS-CoV-2 in Different Types of Clinical Specimens. JAMA.

[58] Zheng S., Fan J., Yu F., Feng B., Lou B., Zou Q., Xie G., Lin S., Wang R., Yang X. (2020). Viral load dynamics and disease severity in patients infected with SARS-CoV-2 in Zhejiang province, China, January-March 2020: Retrospective cohort study. BMJ.

[59] Lamers M.M., Beumer J., van der Vaart J., Knoops K., Puschhof J., Breugem T.I., Ravelli R.B.G., Paul van Schayck J., Mykytyn A.Z., Duimel H.Q. (2020). SARS-CoV-2 productively infects human gut enterocytes. Science.

[60] Jartti T., Hedman K., Jartti L., Ruuskanen O., Allander T., Söderlund-Venermo M. (2012). Human bocavirus—The first 5 years. Rev. Med. Virol..

[61] Kenmoe S., Vernet M.-A., Njankouo-Ripa M., Penlap V.B., Vabret A., Njouom R. (2017). Phylogenic analysis of human bocavirus detected in children with acute respiratory infection in Yaounde, Cameroon. BMC Res. Notes.

[62] Santos N., Peret T.C.T., Humphrey C.D., Albuquerque M.C.M., Silva R.C., Benati F.J., Lu X., Erdman D.D. (2010). Human bocavirus species 2 and 3 in Brazil. J. Clin. Virol..

[63] Han T.-H., Kim C.-H., Park S.-H., Kim E.-J., Chung J.-Y., Hwang E.-S. (2009). Detection of Human Bocavirus-2 in children with acute Gastroenteritis in South Korea. Arch. Virol..

[64] Chow B.D.W., Ou Z., Esper F.P. (2010). Newly recognized bocaviruses (HBoV, HBoV2) in children and adults with gastrointestinal illness in the United States. J. Clin. Virol..

[65] Romani S., Mohebbi S.R., Khanyaghma M., Azimzadeh P., Bozorgi S.M., Damavand B., Jadali F. (2013). Detection of human Bocavirus 1, 2 and 3 from patients with acute gastroenteritis. Gastroenterol. Hepatol. Bed Bench.

[66] De R., Liu L., Qian Y., Zhu R., Deng J., Wang F., Sun Y., Dong H., Jia L., Zhao L. (2017). Risk of acute gastroenteritis associated with human bocavirus infection in children: A systematic review and meta-analysis. PLoS ONE.

[67] Khamrin P., Malasao R., Chaimongkol N., Ukarapol N., Kongsricharoern T., Okitsu S., Hayakawa S., Ushijima H., Maneekarn N. (2012). Circulating of human bocavirus 1, 2, 3, and 4 in pediatric patients with acute gastroenteritis in Thailand. Infect. Genet. Evol..

